# An integrated alarm with clamp system to mitigate vascular access bleeding in hemodialysis: a prospective cohort mixed methods study

**DOI:** 10.1186/s12882-025-04332-8

**Published:** 2025-07-17

**Authors:** Ryan J. Chan, Margaret McGrath-Chong, Christopher T. M. Chan

**Affiliations:** Division of Nephrology, University Health Network, 200 Elizabeth Street 8N Room 846, Toronto, ON M5G 2C4 Canada

**Keywords:** Home hemodialysis, Vascular access, Bleeding, Needle dislodgment, Alarm system

## Abstract

Establishing and maintaining a functional vascular access is essential for patients with end-stage kidney disease on home hemodialysis. Due to patient safety concerns about vascular access complications, particularly needle dislodgement causing bleeding, various technologies have emerged. The purpose of this study was to investigate whether a novel device (developed by Redsense Medical AB) consisting of a sensor placed at the venous puncture site and linked to a base unit with wireless connection to a clamp on the venous line is acceptable from a patient perspective and feasible for implementation in a home hemodialysis program. This was a prospective cohort study of patients undergoing home hemodialysis at the University Health Network in Toronto, Ontario, Canada. We conducted a mixed methods study with a convergent parallel design, collecting both quantitative and qualitative data through two patient questionnaires whereby patients rated their experience with the Redsense system after their first 1–3 treatments and after their last treatment with the system. Thematic analysis was performed with use of open coding and axial coding. Quantitative data was presented as a heat map depicting participants’ scoring of the Redsense system. Differences between the two questionnaires were assessed by Wilcoxon matched-pairs signed rank tests, and the response to select questions were depicted graphically. 21 patients consented to participate in this study, completing a total of 218 dialysis treatments with the Redsense system. Over these treatments, the system was shown to be safe and feasible for patients to use independently at home. The alarm had a relatively elevated false positive rate for both alarm triggering and clamp closure, with this sentiment well described in the qualitative data, as the nuisance of false alarms was a frequent concern described in the post-treatment questionnaire. Patients had other negative comments related to the practicality of the alarm system, the additional burden created, and concern about technical issues relating to wireless technology. Taken together, the data from this study suggest that most patients performing home hemodialysis independently feel that the potential benefits of the Redsense alarm system are outweighed by the additional burden and other negatives associated with the system.

## Clinical trial number

Not applicable.

## Introduction

Establishing a functional vascular access is the fundamental prerequisite in initiating and maintaining a patient with end-stage kidney disease (ESKD) on hemodialysis (HD). Qualified patients may choose home hemodialysis (HHD), particularly given its associated clinical benefits as well as potential for quality of life improvement [1, 2]. HHD training mandates learning strategies to mitigate vascular access complications – including needle taping/fixing, and placement of wetness/enuresis detectors proximal to cannulation sites or on the floor to detect blood leaks [3] – though residual concern persists despite serious bleeding from needle dislodgment being extremely rare events [4].

Various technologies have emerged to address patient safety concerns: one such technology (developed by Redsense Medical AB, Halmstad, Sweden) is a sensor placed at the venous puncture site, and connected to a base unit via optic fiber [5]. The device is activated when > 1 mL of blood comes in contact with the optic fiber, affecting the absorption of light over a specified range of wavelengths for oxygenated to deoxygenated blood, and triggering an alarm in the base unit [5, 6]. The sensor has been shown to be reasonably effective, correctly alarming for 92.5% of blood leakage cases, increasing to 97.2% with the sensor placed near to the puncture site [5].

**Fig. 1:**
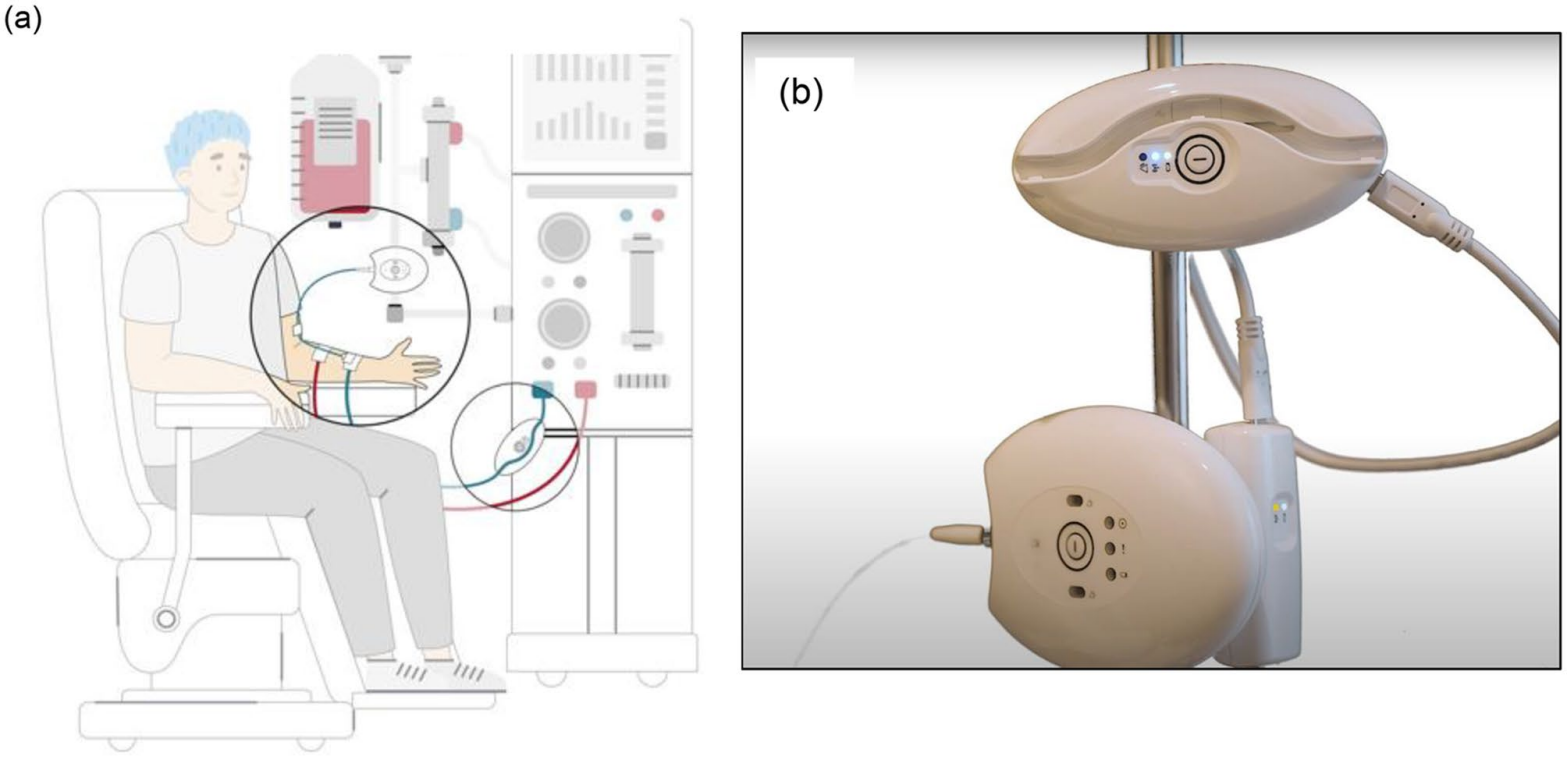
The Redsense blood loss alarm and clamp system. (**a**) Graphical depiction of Redsense alarm system (**b**) Redsense alarm attached to patient IV pole

To mitigate any remaining patient concerns about bleeding or needle dislodgment and allow application regardless of machine and age, a Redsense setup that can automatically (without patient intervention) clamp bloodlines in the event of a blood leak to stop the flow of blood has been designed. A “proof of concept” study confirmed that a clamp on the venous line, operating in conjunction with a venous access blood detector, is feasible regardless of HD location or vascular access type [7]. While the device did improve patients’ safety perception of HD, there was concern about the added burden to patients [7]. In order the improve the user friendliness of the Redsense system, the clamp was updated to have a simple one-button operation, with a wireless connection linking the clamp to the dongle on the alarm unit and allowing for elimination of a cable (Fig. 1). The present study investigates whether this updated Redsense system is acceptable from a patient perspective and feasible for implementation in a home hemodialysis program.

## Materials and methods

### Device design

Redsense is an alarm system that consists of an alarm unit, a fiber optic extension, and a sensor patch. The alarm unit is attached to the patient’s IV pole and connected to a wall outlet via AC adapter. The optical extension fiber is attached to the alarm unit and connected to the fibre from the sensor patch. For patients with an arteriovenous access (fistula or graft), the sensor patch is placed on the patient with the edge of the absorbent foam placed over the insetion point; this is done after the needles have been inserted and before dialysis begins [8]. For patients with a central venous catheter, a larger sensor patch is placed near the blood ports. The alarm unit and clamp are not linked with a cable; they are instead connected to each other via a wireless connection. A graphical depiction of the device setup is shown in Fig. 1, with the different types of alarms described in Appendix A. The product is CE marked and are operated as per the Instructions For Use (IFU) and per its intended use [8].

### Study design

Research Ethics Board and institutional approvals were obtained before initiation of the study (REB #17-5571). This was a prospective cohort study of patients undergoing home hemodialysis at the University Health Network in Toronto, Ontario, Canada. Potential participants were provided with written and oral information about the trial. If they agreed to participate, written informed consent was obtained and a study number was assigned. Participants could freely withdraw from study participation at any time for any reason. Participation in the study did not affect their dialysis prescription or medications.

Staff from Redsense Medical AB., trained five home HD registered nurses and two clinical research coordinators on the use of the Redsense blood loss alarm with clamp. The team, in turn, individually trained each study participant.

We conducted a mixed methods study with a convergent parallel design, collecting both quantitative and qualitative data through two patient questionnaires whereby patients rated their experience with the Redsense system after their first 1–3 treatments (Pre-Survey) and after their last treatment (Post-Survey) with the system [9].

### Data collection

Data were collected and stored using study identification numbers to maintain patient confidentiality. Participants recorded a log of home hemodialysis sessions performed, including all bleeding events, alarms, clamp deployment events, and treatment details.

Participants completed questionnaires regarding their experience with the device after the first 1–3 treatments in the study as well as after the last treatment of the study. Questions were graded from 1 to 5, and related to whether patients believed the Redsense system would lead to safer dialysis, if it was easy to use, if it had affected the treatment positively or negatively, if the alarm would wake them up from sleep, and if it would increase confidence and facilitate the ability to dialyze at home. There was additional space for free text comments and feedback.

### Data analysis

Baseline demographics were described, with medians and interquartile ranges for continuous variables, and numbers and proportions for categorical variables. Qualitative data regarding patient experience with the Redsense system was collected, with thematic analysis performed with use of open coding and axial coding. Quantitative data was presented as a heat map depicting participants’ scoring of the Redsense system in pre- and post-questionnaires. Differences between the two questionnaires were assessed by Wilcoxon matched-pairs signed rank tests, and the response to select questions were depicted graphically.

## Results

**Fig. 2:**
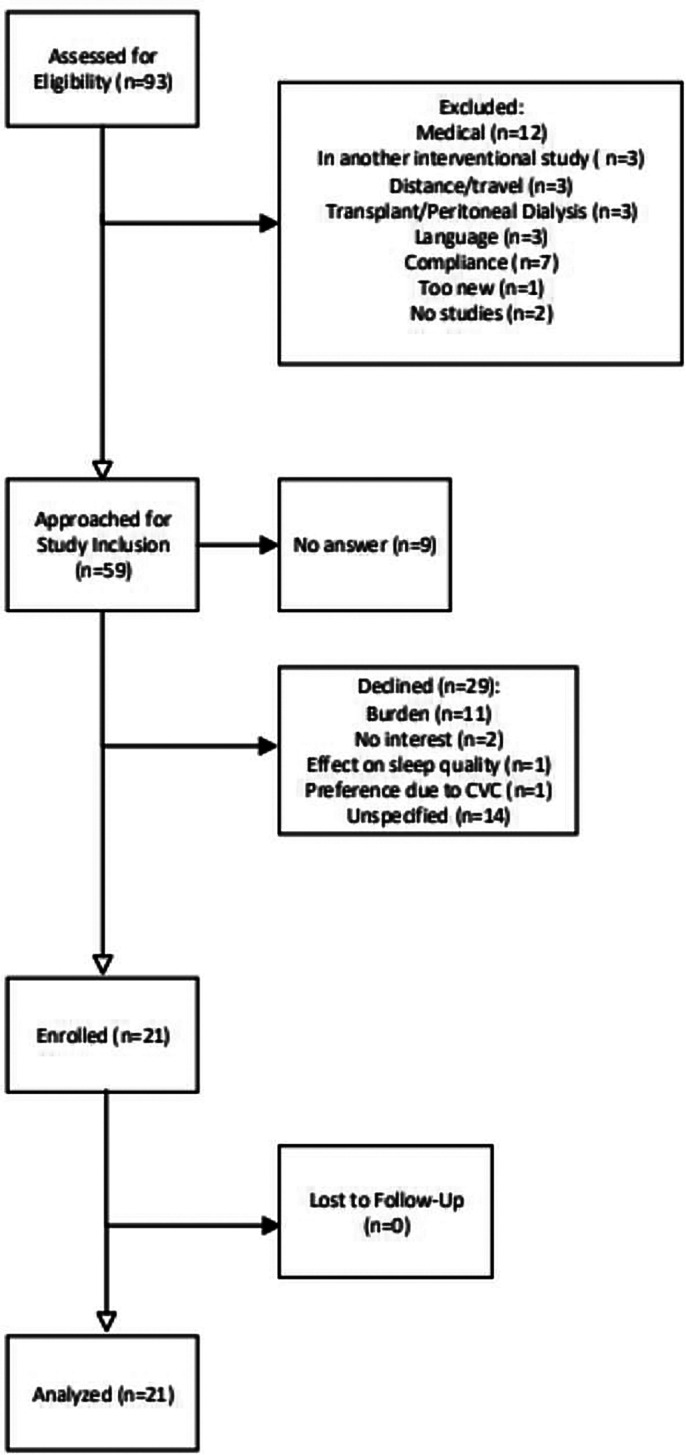
Patient flow

Patient flow is described in Fig. 2.

93 patients were assessed for eligibility. A total of 34 were excluded, and the remaining 59 were approached for study inclusion. 21 patients consented to participate in the study and as there were no patients lost to follow-up, all 21 patients were analyzed.

Baseline demographics for the study participants are described in Table 1.

**Table 1:** Continuous variables are presented as medians with interquartile ranges. Categorical variables are presented as numbers with proportions

Variables	
Male gender, n (%)	18 (86)
Age at recruitment, years	52 (44–60)
Primary Renal Disease, n (%)	
Glomerulonephritis	10 (48)
Polycystic Kidney Disease	4 (19)
Diabetes	1 (5)
Congenital/Hereditary	2 (9)
Renal Vascular	1 (5)
Other	3 (14)
ESRD vintage, years	
Home HD vintage, years	5.9 (2–15)
Previous bleeding event, n (%)	2 (10)
Previous needle dislodgment event, n (%)	4 (19)
Access, n (%)	
Arteriovenous fistula	12 (57)
Arteriovenous graft	8 (38)
Central venous catheter	1 (5)
Dialysis schedule, n (%)	
Nocturnal	18 (86)
Daytime	3 (14)
Medications, n (%)	
Single anti-platelet agent	3 (14)
Dual anti-platelet therapy	0 (0)
Anticoagulant (warfarin or DOAC)	3 (14)
Neither	0 (0)
Spouse or caregiver at home, n (%)	15 (71)

Patients were predominantly male, with a median age of 52 years. Two patients (10%) had had a prior bleeding event and four (19%) had experienced a prior needle dislodgment. Twelve patients (57%) had an arteriovenous fistula, while eight (38%) had a central venous catheter and one (5%) had an arteriovenous graft. There was a wide range of experience with HHD – three patients (14%) had been on HHD for less than one year while five (24%) had been on HHD for over fifteen years; the median number of years on HHD was 5.9 years.

The 21 participants completed a total of 218 dialysis treatments with the Redsense system. Two patients did not report any completed dialysis treatments with the system, five patients had one recorded treatment, while the remaining fourteen greater than one recorded treatment. The median number of treatments completed was 9, and the highest number of completed treatments was 23.

There were no reported bleeding or needle dislodgment episodes over the course of these dialysis treatments (Table 2). There were 40 documented alarms (25 treatments with red alarms, 16 treatments with yellow alarms, 1 treatment with red + yellow alarms) – see Appendix A for description of alarms. There were 15 clamp closure events – all associated with red, yellow, or red + yellow alarms – and 17 instances (6 red, 11 yellow) where there were alarms but clamp did not close.

**Table 2:** Safety data

Event	Count of Reported Events
Bleeding	0
Needle dislodgment	0
Catheter leak	0
Catheter Dislodgment	0

**Fig. 3:**
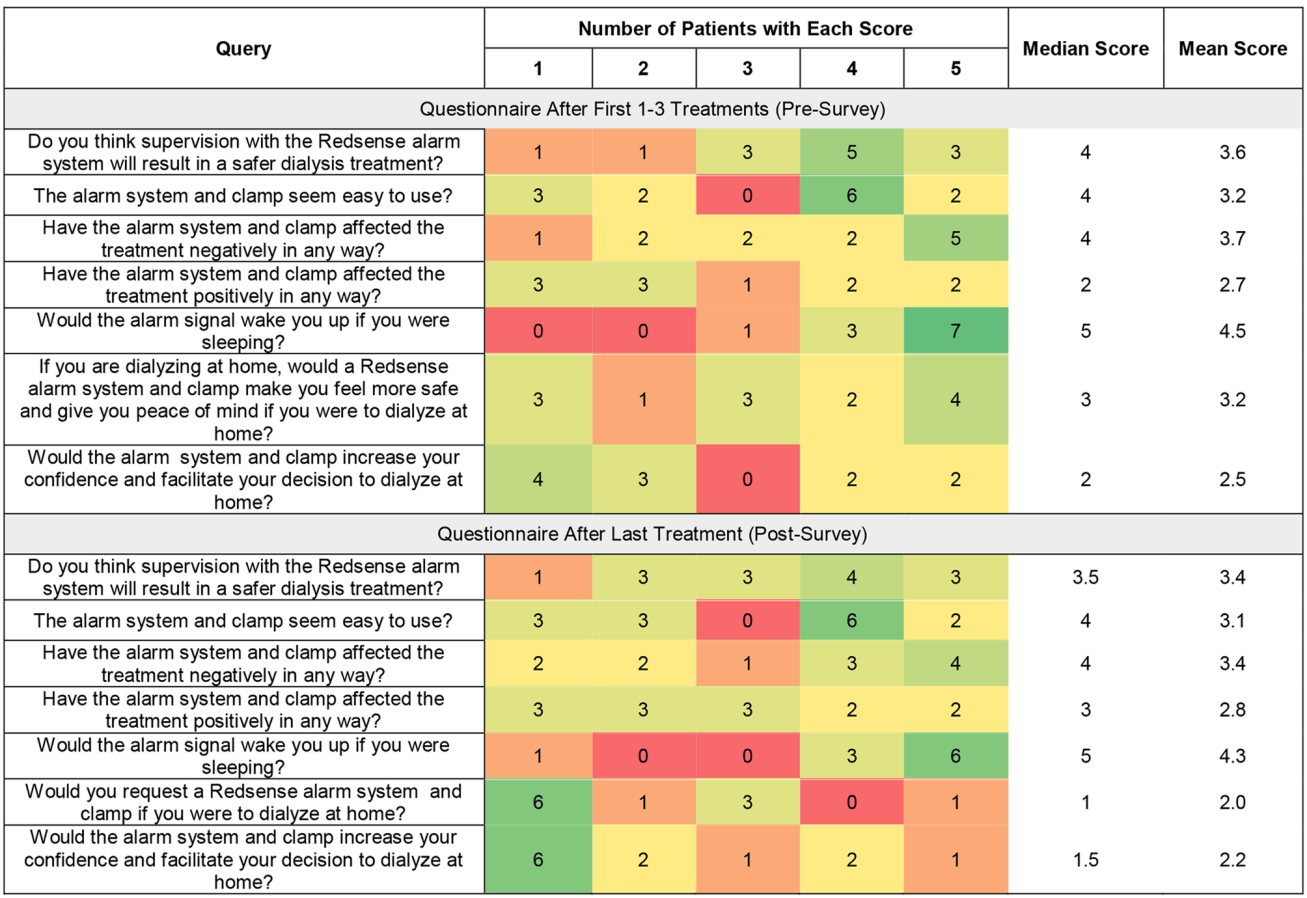
Heat map of scores for pre- and post- questionnaires

Patients completed two surveys, one at baseline (the pre-survey/baseline survey, done after 1–3 treatments with the device), and another after the last treatment of the study (the post-survey). The answers to these survey questions were scored on a scale of 1–5 are summarized in a heat map in Fig. 3.

### Baseline survey

At baseline, the majority of patients felt that supervision with the Redsense alarm system would result a safer dialysis treatment (median score of 4, mean score of 3.6) and most participants felt that the clamp seemed easy to use (median score 4, mean score 3.2). However, participants generally indicated that the alarm system and clamp affected the treatment negatively (median score 4, mean score 3.7) rather than positively (median score 2, mean score 2.7). Patients were split as to whether the Redsense system would make them feel safer or have peace of mind (median score 3, mean score 3.2), and most did not feel that it would increase confidence or facilitate the decision to dialyze at home (median score 2, mean score 2.5). Notably, the four patients who felt they would have increased confidence or the decision to dialyze at home facilitated (score of 4 or 5) were all of more recent HHD vintage (median 11 months since HHD training, versus median 71 months since HHD training in the overall cohort).

### Post-Survey

**Fig. 4:**
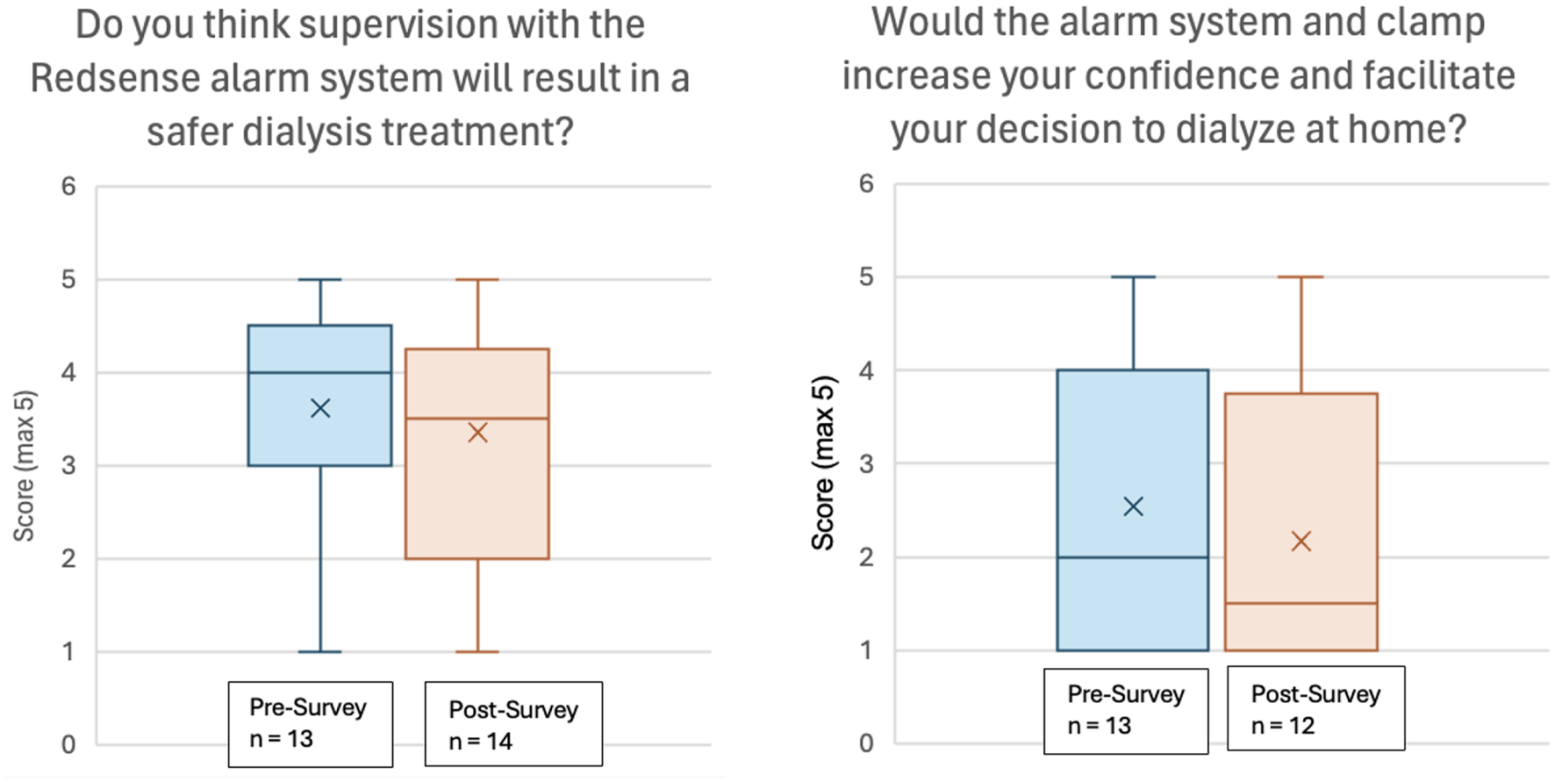
Box and Whisker plots demonstrating median, interquartile range, and range of questionnaire results

**Fig. 5:**
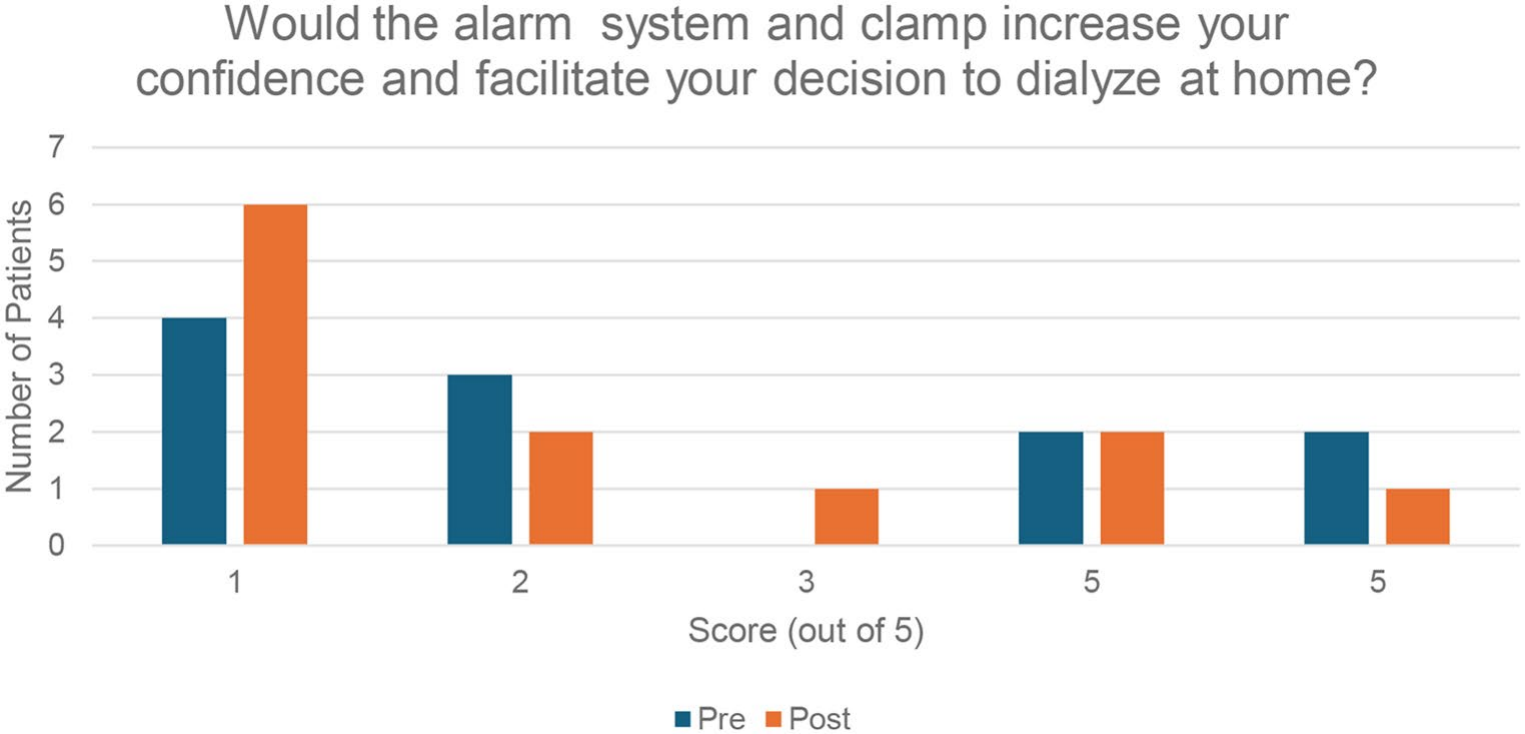
Number of patients indicating increased confidence in dialyzing at home

In the post treatment survey, patients continued to feel that the Redsense alarm system would result in a safer dialysis treatment (median 3.5, mean 3.4), although the degree of positivity was attenuated compared to the pre-survey (median 4, mean 3.6) (Fig. 4). Respondents felt similarly in the post-survey about whether the Redsense system affected the treatment positively or negatively, compared to the pre-survey. There was a decrease in patients’ scores on the post survey for the question asking whether the system increased confidence to dialyze at home, with half of patients (six of twelve) scoring that as a 1 out of 5 (Fig. 5). Of note, the three patients who felt they would have increased confidence or the decision to dialyze at home facilitated (score of 4 or 5) were all of more recent HHD vintage with a median 15 months and mean 14 months since HHD training versus median 71 months and mean of 107 months in the overall cohort. Over half (six of eleven) scored the question asking if they would request a Redsense system if they were to dialyze at home with a 1 out of 5.

Two patients in the cohort had had a previous bleeding event – when asked in the pre-survey about whether they thought the Redsense alarm system would result in a safer dialysis treatment, they gave scores of 4 and 5 (compared to the median score of 4 and mean score of 3.61 in the cohort). In the post-survey, when asked about whether the alarm system and clamp would increase their confidence and facilitate the decision to dialyze at home, these same two patients gave scores of 3 and 5 (compared to the median score of 1.5 and mean score of 2.16 in the cohort).

Qualitative data collected from the pre- and post-questionnaires were classified according to various themes. These are presented alongside illustrative quotations for each identified theme in Table 3. Several patients described an increased sense of safety and confidence in being able to perform their dialysis treatments without complication due to the Redsense alarm system. There were multiple criticisms about the practicality of the system in that it was difficult to set up or use, and that it created an additional burden that added stress and complexity. Many patients expressed being bothered by the frequency of false alarms that disturbed sleep despite no apparent trigger. Finally, there were concerns about technical aspects of the wireless technology adding an extra step to the setup and being a potential source of worry for patients in case the wireless technology were to fail.

**Table 3:** Categories and quotes of perceived factors influencing patient experience

Theme	Illustrative Quotations for Each Identified Theme
*Positive*	
Sense of Safety/Confidence	• “The concept of having a clamp that would stop the blood flow in the event of a disconnection is very reassuring.” • “Having the added safety system would make us more confident to dialyze at home, but wouldn’t have any impact on our decision to do it.” • “I can relax better and sleep better, because I trust the device”
*Negative*	
Practicality	• “One of the most frustrating things about this unit is the lack of information it gives. Trying to decipher what the flashing patterns of 2 LEDs means feels a bity like trying to break a code, in the middle of the night, while you’re groggy from having just been woken up. ...[s]ome form of LCD or even LED display that could give user feedback would make an immense difference. Then as the user we’d know why the unit was alarming and what we needed to do about it.” • “The system and clamp were a pain to use, and too difficult to setup without the aid of a second person helping”
Additional Burden	• “… patients are already stressed with the dialysis machine alone. This adds to the complexity of having to know additional system on top of RO Machine and Dialysis.” • “Don’t like having this extra piece of equipment at night/feels like I have to monitor it to see what’s happening” • “It adds layer of security, but impacts my sleep.”
Nuisance of False Alarms	• “Kept getting alarms and waking me up, even though no blood leak or major movement” • “Nuisance and false alarms stopped treatment unnecessarily, and regularly woke us up, giving us poorer sleeps.”
Technical Issues	• “Wireless tech is just not flawless. Wireless tech also often relies heavily on batteries, adding an extra step to everyday life to use standard things ...[w]henever we do need to use something wireless, there’s always a hardwired backup option on standby for when there’s an issue.”

## Discussion

There are several clinical benefits to home hemodialysis, but these benefits must be balanced against the risks of the vascular access used to dialyze independently [10]. The ideal vascular access provides reliable, complication-free ability to deliver prescribed dialysis [11, 12]. However, one of the most significant concerns with performing HHD is trepidation relating to needle dislodgment and subsequent bleeding. This is particularly notable for venous dislodgment (VND) – with typical blood flow (Qb) rates of 300–500 mL/min, VND could lead to a patient losing upwards 2 L of blood (from an average human blood volume of 5 L) within only minutes, and precipitate development of hemorrhagic shock [13], especially if the bleeding goes unnoticed due to the patient sleeping or the access being obscured. Importantly, over 70% of patients have venous access pressures too small to trigger a machine alarm in the case of a VND [14], and if there is an upward drift in the venous pressure (due to ultrafiltration increasing a patient’s hematocrit and blood viscosity), the pressure drop required to trigger an alarm is increased and heightens the risk of undetected VND [15]. Therefore, a large focus of HHD patient training relates to strategies to minimize the risk of bleeding or mitigate morbidity should bleeding occur [12].

The adverse event rate for patients on home hemodialysis is extremely low, as demonstrated by a retrospective cohort study of 202 HHD patients which reported a serious adverse event rate of only 0.009 per patient-year of HHD and 0.038 per 1,000 dialysis treatments [4]. This study examined 183,603 dialysis treatment and found a total of 18 needle dislodgments, of which 14 (78%) were attributed to patient error [4]. A similar study in two Canadian HHD programs reviewed 500 patient-years of dialysis, identifying six potentially fatal events and reporting a major adverse event rate of 0.06 per 1000 dialysis treatments [16]. Despite the overall low risk of serious adverse events, patient concerns about the safety of utilizing a dialysis vascular access independently at home remain a major barrier to choosing home hemodialysis [17–19].

Addressing patient error or adverse events through human factors engineering [20] may help to reduce complications and increase the safety perception of home hemodialysis. A prior study of the Redsense system had found the system to be functional, but raised concerns about the additional burden created and impact on user acceptability [7]. In this study, we examined the updated Redsense system, which consists of an one-button alarm unit wirelessly connected to a clamp that could stop the flow of blood if a blood leak was detected.

A previously published study on an older version of the Redsense system found that it had correctly identified all three bleeding events over the 214 HHD treatments in the study period [7]. In our study, the device sensitivity in identifying bleeding events and triggering the venous line clamp could not be properly assessed, as there were no reported events (bleeding, needle or catheter dislodgment, or catheter leak) for the 218 treatments within the study period and therefore no true positives or false negatives. Despite this, the alarm was triggered 40 times and there were 15 clamp closure events, representing a relatively elevated false positive rate for both alarm triggering and clamp closure (especially considering that there were only five false positive clamp events for 214 treatments in the prior study [7]). This sentiment was also well described in the qualitative data, as the nuisance of false alarms was a frequent concern described in the post-treatment questionnaire; this was compounded by the fact that there were no true positive adverse events during the study period and the incidence rate of a severe adverse event in the literature is extremely low, thus making the purported benefit of the system less tangible. The reason for the high rate of false alarms was not recorded, but we speculate that moisture at the site was the main cause for a false positive.

In addition to frustrations about the occurrence of false alarms, patients had other negative comments related to the practicality of the alarm system, the additional burden created, and concern about technical issues relating to wireless technology (Table 3). This manifested quantitatively as low scores in the post-survey for questions on whether patients would request the alarm system and clamp for HHD (median score 1, mean score 2.0) or whether it would increase confidence and facilitate the decision to do HHD (median score 1.5, mean score 2.2), both lower than the corresponding questions in the pre-survey. Taken together, the data from this study suggest that most patients performing HHD at home independently feel that the potential benefits of the Redsense alarm system are outweighed by the additional burden and other negatives associated with the system.

There were select patients who felt positively about the Redsense alarm system with clamp. For instance, patients of more recent HHD vintage tended to view the system more positively, evidenced by the fact that the three patients who had increased confidence of the decision to dialyze at home were of relatively more recent HHD vintage. The two patients in the cohort who had had a prior bleeding event also tended towards higher scores for the same question in both the pre- and post-surveys. Therefore, in spite of its challenges in widespread use for the general HHD population – as evidenced by the low number of patients who indicated that the Redsense system affected the treatment positively, that they would request the system if dialyzing at home, or that it would increase confidence – there could be potential situations where the system could be useful. For instance, the data suggest that the alarm system and clamp may be useful for patients who have had a prior bleeding event and wish to have the peace of mind afforded by the system. Similarly, patients of more recent vintage or those unfamiliar with a hemodialysis vascular access but considering HHD as a modality may be reassured by the presence of the system.

The findings of our study have limited generalizability given that there were no vascular access bleeding events during the study period, precluding our ability to assess the sensitivity of the Redsense system. Despite there being 218 dialysis sessions recorded through the study period, these were distributed unevenly amongst the study participants, as one third (7 of 21 patients) had only one recorded treatment session or less reported – this could have further skewed our data and limits interpretation. While there was a signal towards certain populations perceiving the system more favorably (those who started HHD more recently, those with a prior access bleeding event), the absolute number of patients in these groups was small, making these findings exploratory.

In closing, we described a blood loss alarm system with wirelessly connected clamp designed to increase the safety of hemodialysis treatments. Over 218 treatments, the system was shown to be safe and feasible for patients to use independently at home. Though iterative design modifications have been made compared to prior versions of the system, there remain several challenges to its broad useability and acceptability amongst the general HHD population. Despite this, the technology has potential utility for targeted populations, and future studies could explore if patients in other health care settings could benefit from this type of semi-autonomous system.

## Appendix A

**Table:** 

Alarm Unit	Indicator	Significance
*Pre-HD*	Flashing Battery indicator Light	Alarm unit charging
	Solid indicator light	Charging complete
	Yellow indicator light	Any of: Moisture in contact with sensor Sensor loose, not connected, or broken Optical extension fiber not connected correctly
	Flashing green indicator light	Alarm performing self-test
*During HD*	Continuous green indicator light	Unit running
	Yellow warning indicator light (followed by intermittent alarm signal)	Any of: Moisture in contact with sensor Sensor loose, not connected, or broken Optical extension fiber not connected correctly
	Red (followed by continuous alarm signal)	Blood detected by sensor, either due to venous needle dislodgment or leak
	All indicator lights flashing	Internal error detected – contact Redsense Medical for support
**Bloodline Clamp**	**Indicator**	**Significance**
Communication	Fast blue blink	Bloodline Clamp is not paired or connected to Dongle, or wireless connection to Dongle lost
	Slow blue blink	Normal – Bloodline Clamp is paired and connected to a Dongle and treatment ongoing
	Yellow blink	Radio contact with Dongle is lost for more than one (1) minute
	Alternating blue and yellow	The user has pressed the buttn on the Bloodline Clamp to start connection check with the Dongle
Warning or Alarm	Red → Warning	Bloodline Clamp has received blood leakage detection alarm from Alarm Unit and the bloodline is clamped
	Red blink → high priority alarm condition	Bloodline Clamp has detected internal error

## Data Availability

The datasets used and/or analysed during the current study are available from the corresponding author on reasonable request.
